# Current Status of Multidisciplinary Treatment Strategies for Hepatocellular Carcinoma in the Era of Advanced Systemic Therapies

**DOI:** 10.1002/ags3.70153

**Published:** 2025-12-22

**Authors:** Keiichi Akahoshi, Shun Kaneko, Shinji Tanaka, Minoru Tanabe, Daisuke Ban

**Affiliations:** ^1^ Department of Hepatobiliary and Pancreatic Surgery Institute of Science Tokyo Tokyo Japan; ^2^ Department of Gastroenterology and Hepatology Institute of Science Tokyo Tokyo Japan; ^3^ Department of Molecular Oncology Institute of Science Tokyo Tokyo Japan; ^4^ Department of Surgery Kashiwa Municipal Hospital Tokyo Japan

**Keywords:** borderline resectable, conversion surgery, hepatocellular carcinoma, oncological resectability criteria, systemic therapy

## Abstract

The therapeutic landscape of hepatocellular carcinoma (HCC) has been transformed by recent advancements in systemic therapies, particularly with the introduction of immune checkpoint inhibitors, expanding treatment options beyond conventional locoregional approaches. This review provides an overview of evidence accumulated from recent Phase III trials of first‐line regimens and key second‐line agents and examines how these advances enable multidisciplinary treatment strategies and timely transition to curative local treatments. We highlight prospective and retrospective data on systemic therapy administered in combination with or in sequence with locoregional treatment modalities, including TACE‐based combinations and “conversion” concepts leading to resection. A central focus is the oncological resectability criteria proposed by the Japan Liver Cancer Association and the Japanese Society of Hepato‐Biliary‐Pancreatic Surgery, which provide an objective framework to assess surgical indications under contemporary systemic therapy. Validation studies have consistently demonstrated robust prognostic stratification across resectable (R), borderline resectable 1 (BR1), and borderline resectable 2 (BR2) categories. Evidence for application of the oncological resectability criteria in treatment decision‐making is still insufficient. Thus, future prospective studies and real‐world registries aligned with the resectability framework are essential for defining the optimal timing, sequencing, and candidacy for surgery to ultimately enable provision of individualized, evidence‐based care for patients with advanced HCC.

## Abstract 2

Recent advances in immune checkpoint inhibitor‐based systemic therapies have transformed the treatment landscape of hepatocellular carcinoma, enabling multidisciplinary strategies that integrate systemic therapy with curative local treatments. This review summarizes the latest Phase III evidence and examines how emerging concepts—particularly the oncological resectability criteria (R/BR1/BR2)—provide an objective framework for selecting surgical candidates in the era of advanced systemic therapies. We also highlight current limitations and future directions, including the need for prospective validation and optimized timing and patient selection for surgery following systemic therapy.

## 1 Introduction

Hepatocellular carcinoma (HCC) is one of the most significant global health concerns, accounting for approximately 90% of all primary liver cancers. Now, liver cancer ranks as the sixth most commonly diagnosed cancer and the third leading cause of cancer‐related mortality worldwide [1]. Since 2018, the therapeutic landscape for unresectable HCC has improved dramatically, driven mainly by the development of novel systemic therapies, including molecular‐targeted agents and immune checkpoint inhibitors (ICIs) [2]. These treatments have not only yielded significant improvements in survival outcomes but also broadened the therapeutic options beyond conventional locoregional methods like transarterial chemoembolization (TACE). Many internationally adopted treatment algorithms, including the management guideline proposed by the Barcelona Clinic Liver Cancer (BCLC) staging system, have been recently updated/revised to reflect these advances. For example, while multinodular HCC was previously managed solely by TACE under the BCLC 2018 framework, the 2022 update incorporates systemic therapy and liver transplantation as additional options [3, 4].

In real‐world clinical practice, surgical resection following tumor downstaging with systemic therapy, while previously considered as being exceedingly rare, has become increasingly adopted [5, 6, 7]. This emerging strategy highlights the evolving relevance of multidisciplinary approaches that integrate systemic and local treatments to enhance the oncologic outcomes of advanced HCC. To provide a common language and framework for assessing surgical indications in this evolving context, the Japan Liver Cancer Association (JLCA) and the Japanese Society of Hepato‐Biliary‐Pancreatic Surgery (JSHBPS) have jointly proposed oncological resectability criteria for HCC [8].

In this review, we attempt to summarize the recent progress in systemic therapy for HCC and to explore the current status and challenges of multidisciplinary treatment strategies for advanced HCC, with particular emphasis on the conceptual foundation and clinical usefulness of the proposed oncological resectability criteria.

## 2 Current Systemic Therapies for HCC

Until 2008, there were no effective systemic therapies for patients with advanced‐stage HCC. Approval for sorafenib in 2009 marked a groundbreaking milestone in the treatment of HCC [9]. However, in the decade that followed, while numerous clinical trials were conducted focusing primarily on multi‐tyrosine kinase inhibitors such as sunitinib and brivanib, none demonstrated sufficient efficacy of any agents that could be adopted as first‐line systemic therapy for HCC [10, 11]. Subsequently, since the approval of lenvatinib in 2018, the field of systemic therapy has advanced dramatically with the introduction and approval of not only additional molecular‐targeted agents but also of several ICIs, transforming the treatment landscape for unresectable HCC [12]. Results of pivotal Phase III clinical studies are summarized in Table 1 and Table S1.

**TABLE 1:** Results of pivotal phase III clinical studies of systemic therapies for hepatocellular carcinoma.

Trial name	Year	Design	Number (total)	Etiology (HBV/HCV/other)	Study arm	Control arm	Median OS (months)	Hazard ratio for OS (95% CI)	*p*	Median PFS (months)	Time to response (months) (RECIST 1.1)	Time to response (months) (mRECIST)
SHARP	2008	Phase III, RCT	602	18%/28%/53%	Sorafenib 400 mg twice daily	Placebo	10.7 vs. 7.9	0.69 (0.55–0.87)	< 0.001	5.5 vs. 2.8	NR	NR
REFLECT	2018	Phase III, non‐inferiority	954	50%/23%/27%	Lenvatinib (weight‐based) daily	Sorafenib	13.6 vs. 12.3	0.92 (0.79–1.06)	Non‐inferiority met	7.3 vs. 3.6	2.8 vs. NR	1.9 vs. NR
IMbrave150	2020	Phase III, superiority	501	48%/22%/31%	Atezolizumab 1200 mg + bevacizumab 15 mg/kg q3w	Sorafenib	19.2 vs. 13.4	0.66 (0.52–0.85)	< 0.001	6.9 vs. 4.3	2.8 vs. 2.6	2.8 vs. 3.3
HIMALAYA	2022	Phase III, superiority	782	31%/27%/42%	Tremelimumab 300 mg once + durvalumab 1500 mg q4w (STRIDE)	Sorafenib	16.4 vs. 13.8	0.78 (0.65–0.93)	0.0035	3.8 vs. 4.1	2.2 vs. 3.8	NR
CheckMate 9DW	2025	Phase III, superiority	668	34%/28%/36%	Nivolumab 1 mg/kg + ipilimumab 3 mg/kg q3w × 4 doses	Lenvatinib or sorafenib	23.7 vs. 20.6	0.79 (0.65–0.96)	0.018	9.1 vs. 9.2	2.2 vs. 3.7	NR

Abbreviations: CI, confidence interval; HBV, hepatitis B virus; HCV, hepatitis C virus; mRECIST, modified Response Evaluation Criteria in Solid Tumors; NR, not reported; OS, overall survival; PFS, progression‐free survival; RCT, randomized controlled trial; RECIST, Response Evaluation Criteria in Solid Tumors; STRIDE, single tremelimumab regular interval durvalumab.

### 2.1 First‐Line Systemic Therapies

#### 2.1.1 Sorafenib

Sorafenib, a representative treatment agent for HCC, is an oral multi‐tyrosine kinase inhibitor that exerts its antitumor effects by inhibiting various kinases, including Raf, VEGFR, and PDGFR. Two large‐scale clinical trials conducted in the 2000s, the SHARP trial and the Asia‐Pacific trial, demonstrated significant prolongation of the overall survival (OS) in the sorafenib arm as compared with the placebo arm, which led to approval of the drug for the treatment of HCC in 2009. While the introduction of sorafenib was groundbreaking, the objective response rate (ORR) was only 2%, and the drug had relatively strong side effects, including hand‐foot syndrome and gastrointestinal symptoms [9, 13]. This highlighted the need for more effective and less toxic drugs.

#### 2.1.2 Lenvatinib

In 2018, the Phase III REFLECT trial, which compared sorafenib with lenvatinib, yielded encouraging results. In this trial, patients with unresectable HCC who had not received prior systemic therapy were randomized at a ratio of 1:1 into the sorafenib and lenvatinib arms. The OS was 12.3 months in the sorafenib arm and 13.6 months in the lenvatinib arm, with a hazard ratio (HR) of 0.92 (0.79–1.06), indicating non‐inferiority of lenvatinib to sorafenib, as the 95% CI was below the non‐inferiority margin of 1.08 (Table 1) [12]. Notably, the lenvatinib arm showed a high response rate: the ORR, obtained by summing up the complete response (CR) and partial response (PR) rates, was 18.8% (RECIST ver1.1) in the lenvatinib arm as compared with 6.5% in the sorafenib arm. When assessed using the mRECIST criteria, the ORR in the lenvatinib arm increased to 40.6%, significantly higher than that in the sorafenib arm of 12.4% (Table S1). Based on the results of the REFLECT trial, lenvatinib was approved in 2018 as a first‐line treatment agent for unresectable HCC, marking a significant advancement in systemic therapy for HCC. Common adverse effects of lenvatinib include hand‐foot syndrome, gastrointestinal symptoms, hypertension, proteinuria, and thyroid dysfunction.

#### 2.1.3 Atezolizumab Plus Bevacizumab

One of the most notable recent advancements in cancer treatment has been the introduction of ICIs. The combined immune checkpoint inhibitor regimen of atezolizumab, an anti‐PD‐L1 antibody, plus bevacizumab, an anti‐VEGF‐A antibody, exhibited superior efficacy as compared with sorafenib in the Phase III IMbrave150 trial [14]. PD‐L1, expressed on the tumor cells, binds to PD‐1 expressed on T cells, suppressing their activation and allowing the tumor to evade immune responses. Atezolizumab blocks this PD‐L1/PD‐1 pathway, reactivating and enhancing the antitumor effects of T cells [15]. Bevacizumab inhibits VEGF‐mediated suppression of dendritic cell maturation, thereby improving the immunosuppressive tumor microenvironment and promoting CD8+ T cell infiltration into tumors. It also exerts direct antitumor effects by inhibiting tumor angiogenesis [16]. The IMbrave150 trial showed superior OS and progression‐free survival (PFS) in the atezolizumab plus bevacizumab arm as compared with that in the sorafenib arm. The median OS was not reached in the atezolizumab plus bevacizumab arm, whereas it was 13.2 months (95% CI, 10.4–not estimable [NE]) in the sorafenib arm, with an HR of 0.58 (95% CI, 0.42–0.79; *p* < 0.001) [14]. Following publication of these results, the combination regimen of atezolizumab plus bevacizumab was approved as a first‐line systemic therapy regimen for unresectable HCC in September 2020. Updated analyses showed an OS of 19.2 months (95% CI, 17.0–23.7) in the atezolizumab plus bevacizumab arm as compared with that of 13.4 months (95% CI, 11.4–16.9) in the sorafenib arm, with an HR of 0.66 (95% CI, 0.52–0.85; *p* < 0.001). The PFS was 6.9 months (95% CI, 5.7–8.6) in the atezolizumab plus bevacizumab arm versus 4.3 months (95% CI, 4.0–5.6) in the sorafenib arm, with an HR of 0.65 (95% CI, 0.53–0.81; *p* = 0.0001). The ORR (RECIST ver1.1) was 27.3% in the atezolizumab plus bevacizumab arm as compared with 11.9% in the sorafenib arm, establishing the former as the preferred first‐line treatment [17]. However, immune‐related adverse events (irAEs) specific to ICIs and hemorrhagic events associated with bevacizumab began to draw attention. Therefore, patients should be carefully evaluated for autoimmune diseases and bleeding risks, such as esophageal varices, before this treatment is initiated.

#### 2.1.4 Durvalumab Plus Tremelimumab

This combination immunotherapy regimen is composed of durvalumab, an anti‐PD‐L1 antibody, and tremelimumab, an anti‐CTLA‐4 antibody. Tremelimumab targets CTLA‐4 on T cells, blocking its function to enhance T cell activation, boost the immune response against cancer, and induce cancer cell death. The regimen, also known as STRIDE (single tremelimumab, regular‐interval durvalumab), involves a single high‐dose administration of tremelimumab at the start [18]. The Phase III HIMALAYA trial compared durvalumab plus tremelimumab, durvalumab monotherapy, and sorafenib monotherapy as first‐line treatment in patients with unresectable HCC [19]. The OS in the durvalumab plus tremelimumab arm was 16.4 months (95% CI, 14.2–19.6) as compared with 13.8 months (95% CI, 12.3–16.1) in the sorafenib arm, with an HR of 0.78 (95% CI, 0.65–0.93), demonstrating superiority of the former (Table 1). The ORR (RECIST ver1.1) to the combination regimen was also significantly higher at 20.1% as compared with 5.1% in the sorafenib arm (Table S1). As a result, durvalumab monotherapy was approved in December 2022, followed by approval of the durvalumab plus tremelimumab combination regimen in March 2023 as first‐line treatment for unresectable HCC. Combined immunotherapy regimens are associated with a higher incidence of irAEs as compared to monotherapies, with 20.1% of patients requiring steroid treatment [19]. However, they are a feasible option for patients at risk of bleeding events (e.g., esophageal varices) or proteinuria, commonly seen in HCC cases.

#### 2.1.5 Nivolumab Plus Ipilimumab

The combination regimen of Nivolumab (anti‐PD‐1 antibody) plus Ipilimumab (anti‐CTLA‐4 antibody) was evaluated as a first‐line treatment for unresectable HCC in the global phase III CheckMate 9DW trial [20]. This open‐label randomized study compared the efficacy and safety of four doses of nivolumab (1 mg/kg) plus ipilimumab (3 mg/kg) administered every 3 weeks, followed by a maintenance dose of nivolumab, against the investigator's choice of lenvatinib or sorafenib. This pivotal trial was recently published in *The Lancet* in May 2025. The combination therapy significantly improved the OS, with a median OS of 23.7 months (95% CI, 18.8–29.4) versus 20.6 months (95% CI, 17.5–22.5) in the control arm, yielding an HR of 0.79 (95% CI, 0.65–0.96; *p* = 0.018). Importantly, 85% of patients in the control group received lenvatinib, indicating that this result predominantly demonstrates a survival benefit over lenvatinib rather than sorafenib (Table 1). The ORR based on RECIST v1.1 was 36% in the nivolumab plus ipilimumab arm versus 13% in the control arm. Notably, the median time to response (TTR) was shorter (2.2 months vs. 3.7 months) and the median duration of response was remarkably longer (30.4 months vs. 12.9 months) in the combination arm, suggesting both rapid and sustained tumor control (Table 1; Table S1). As expected with dual ICI, the incidence of irAEs was higher in the combination arm: 58% of patients experienced immune‐mediated adverse events of any grade, and 28% developed grade 3–4 events. Treatment‐related deaths occurred in 12 patients who received nivolumab plus ipilimumab, most commonly due to immune‐mediated hepatitis and liver‐related complications [20]. Despite these safety concerns, the robust survival benefit and sustained responses lent support to the use of Nivolumab plus ipilimumab as a promising new first‐line option, and this regimen was approved in June 2025 in Japan.

### 2.2 Second‐Line Systemic Therapies

Three agents, regorafenib, ramucirumab, and cabozantinib, have been approved as second‐line therapies for use after sorafenib treatment. Regorafenib is an oral multi‐tyrosine kinase inhibitor that is structurally similar to sorafenib. Its efficacy was demonstrated in the Phase III RESORCE trial, which was conducted in patients with unresectable HCC who showed disease progression after first‐line Sorafenib, leading to its approval in 2017 [21]. Ramucirumab is a human monoclonal antibody targeting VEGFR‐2, administered biweekly via intravenous infusion. Its efficacy was demonstrated in the Phase III REACH‐2 trial, which included HCC patients with serum AFP levels of ≥ 400 ng/mL who were resistant or intolerant to sorafenib, leading to its approval in 2019 [22]. Cabozantinib is another oral multi‐tyrosine kinase inhibitor. Its efficacy was demonstrated in the Phase III CELESTIAL trial, which targeted patients who had received 1–2 prior systemic therapies, including sorafenib, but exhibited disease progression. It was approved in 2020 [23]. These therapies represent significant advancements in the management of unresectable HCC, offering new hope and improved outcomes for patients.

Recently, treatment sequencing after atezolizumab plus bevacizumab failure has emerged as an important clinical issue. Real‐world and multicenter cohort studies have shown that lenvatinib, regorafenib, and cabozantinib remain feasible and effective second‐line options, achieving ORR of approximately 10%–20% and median OS of 13–15 months. In contrast, ICI re‐challenge has shown limited but potentially meaningful activity in selected patients. However, these findings are based on analyses of a small population, and the overall level of evidence remains very limited [24]. Therefore, TKIs currently serve as the mainstay of post‐atezolizumab plus bevacizumab therapy, while further prospective studies are warranted to establish optimal sequencing strategies and clarify the role of ICI re‐challenge.

## 3 Advances in Systemic Therapy and Directions of Multidisciplinary Treatment

Since 2001, when the European Association for the Study of the Liver (EASL) published its HCC management guidelines [25], more than 20 guidelines have been published, with some differences in surgical indications among the guidelines [26]. These differences in the surgical indications reflect variations in transplant donor availability, healthcare infrastructure, and historical medical practices among countries [27]. However, tumor conditions that fall into the gap between the guideline criteria, such as tumors that are technically resectable but oncologically borderline, are often associated with a high risk of early recurrence, even when macroscopic curative resection has been achieved. These instances highlight the limitations of single‐modality therapy and underscore the growing importance of integrated, multidisciplinary treatment strategies. Advances in systemic therapy, particularly the advent of therapy with ICIs and molecular‐targeted agents, have prompted renewed discussions from both medical and surgical communities on how to optimize the sequence and combination of systemic and locoregional modalities. These efforts have led to a paradigm shift in the management of unresectable HCC, with emphasis placed on achieving long‐term disease control, and where possible, on transition to potentially curative interventions. Table 2 summarizes the key prospective clinical trials that evaluated such multimodal strategies integrating systemic therapy with locoregional or surgical treatments.

**TABLE 2:** Pivotal prospective clinical trials of combined systemic therapy plus locoregional or surgical treatments for hepatocellular carcinoma.

Trial name	Study design	Patient population	Regimen	Primary endpoint	Resection rate	Key findings	Status
TACTICS	Phase II, RCT	Unresectable HCC	TACE plus sorafenib vs. TACE alone	PFS	—	Improved PFS, no OS benefit	Published
TACTICS‐L	Phase II, Single‐arm	Unresectable HCC	TACE plus lenvatinib	PFS by RECICL	—	ORR 88.7%, CR 67.7%	Published
LAUNCH	Phase III, RCT	Advanced HCC (MVI, EHS included)	Lenvatinib plus TACE vs. lenvatinib alone	OS	—	HR 0.45 (OS), HR 0.43 (PFS)	Published
LEAP‐012	Phase III, RCT	Unresectable, non‐metastatic HCC	Lenvatinib + pembrolizumab + TACE vs. TACE	PFS, OS	—	PFS ↑ (HR 0.66), OS not yet significant	Published
TALENTACE	Phase III, RCT	TACE‐eligible, systemic therapy–naïve HCC	Atezolizumab + bevacizumab + TACE vs. TACE	TACE‐PFS, OS	—	Improved TACE‐PFS	Ongoing
EMERALD‐3	Phase III, RCT	HCC not amenable to curative therapy but eligible for TACE	Durvalumab + tremelimumab + TACE (±lenvatinib) vs. TACE	PFS	—	Ongoing	Ongoing
LENS‐HCC	Phase II, single‐arm	Unresectable HCC	Lenvatinib followed by surgery	Resection rate	67.3%	Favorable resection rate and 1‐year OS	Published
RACB	Phase II, single‐arm	Unresectable HCC	Atezolizumab + bevacizumab followed by surgery	PFS	48% (R0: 87.5%)	Feasible with no perioperative mortality	Ongoing
LEOPARD‐NEO	Phase II, single‐arm	BR‐HCC with vascular invasion	Lenvatinib + HAIC followed by surgery	Resection rate		Ongoing	Ongoing
Cabozantinib + nivolumab	Phase Ib, single‐arm	Locally advanced HCC	Cabozantinib + nivolumab	Safety and feasibility	80% (12/15)	42% major pathological response	Published

Abbreviations: CR, complete response; EHS, extrahepatic spreads; HCC, hepatocellular carcinoma; HR, hazard ratio; MVI, macrovascular invasion; ORR, objective response rate; OS, overall survival; PFS, progression‐free survival; RCT, randomized controlled trial.

### 3.1 Combined TACE and Systemic Therapy

TACE remains the mainstay of treatment for intermediate‐stage HCC. However, repeated TACE procedures are associated with progressive liver dysfunction and limited long‐term efficacy, especially in patients with tumors that are large, infiltrative, or poorly differentiated. Several studies have therefore investigated the effectiveness of combining TACE with systemic therapy. The TACTICS trial demonstrated a significant improvement in the PFS when sorafenib was combined with on‐demand TACE, although no OS benefit was observed [28, 29]. The TACTICS‐L trial built upon this with lenvatinib, showing a high response rate (ORR 88.7%, CR 67.7%) by RECICL [30]. In the phase III LAUNCH trial, lenvatinib plus TACE yielded significantly improved OS as well as PFS as compared with lenvatinib alone; the median OS was significantly longer in the lenvatinib plus TACE arm (17.8 months vs. 11.5 months; hazard ratio, 0.45; *p* < 0.001). The median PFS was 10.6 months in the lenvatinib plus TACE arm and 6.4 months in the lenvatinib‐alone arm (HR, 0.43; *p* < 0.001) [31]. More recently, the LEAP‐012 trial demonstrated a significantly better PFS in the TACE plus pembrolizumab + lenvatinib arm (median, 14.6 months) than in the TACE + placebo arm (median, 10.0 months) (HR 0.66; 95% CI, 0.51–0.84; *p* = 0.002). In addition, a favorable OS trend (HR 0.80; 95% CI, 0.57–1.11; *p* = 0.0867) and a significantly better ORR (TACE plus pembrolizumab + lenvatinib, 71.3%; TACE + placebo, 49.8%; *p* < 0.0001) were also observed [32]. Based on these findings, multiple phase III trials are now ongoing, including the TALENTACE trial, comparing control with TACE + atezolizumab + bevacizumab, and the EMERALD‐3 trial comparing control with TACE + STRIDE with or without lenvatinib [33, 34, 35]. The primary agents used in recent studies have been ICIs, which are considered the most likely to activate the immune system when administered with TACE, resulting in synergistic effects and enhanced antitumor efficacy.

### 3.2 ABC Conversion Therapy and Locoregional Integration

The concept of ABC conversion therapy, namely, atezolizumab plus bevacizumab therapy followed by curative treatment, has emerged as an innovative approach for patients with intermediate‐stage HCC who are deemed unsuitable candidates for TACE. When tumor shrinkage is obtained with atezolizumab plus bevacizumab, curative conversion by resection, ablation, or superselective TACE makes it possible to achieve CR [36]. A multicenter proof‐of‐concept study reported that 35% of patients achieved clinical or pathological CR after receiving atezolizumab plus bevacizumab; of these, 66% reached a drug‐free status following curative treatment [37].

These results suggest that systemic therapy may serve as a bridge to curative local treatment, and that a timely transition might be beneficial in selected patients rather than continuing systemic therapy until disease progression. The phase III IMPACT study is currently underway to validate this approach. In this study, patients are stratified after 12 weeks of induction therapy with atezolizumab plus bevacizumab into two cohorts: a randomized cohort of patients who achieved SD, or a cohort of patients who achieved CR or PR in which atezolizumab plus bevacizumab therapy was followed by curative conversion (ABC‐conversion). Patients in the randomized cohort are receiving atezolizumab plus bevacizumab and intrahepatic control TACE (Group A) or are being continued on atezolizumab plus bevacizumab (Group B). All cohorts may be evaluated for curative conversion therapies targeting residual tumors, provided these therapies are deemed to have curative potential. The primary endpoint is OS in the randomized cohort and conversion rate in the ABC‐conversion cohort [38]. The IMPACT study is anticipated to aid in establishing a response‐guided treatment strategy for unresectable HCC by identifying the optimal treatment based on the therapeutic response to atezolizumab plus bevacizumab.

### 3.3 Surgical Resection Following Systemic Therapy

Recent advances in systemic therapy have led to a paradigm shift in the management of HCC, especially for patients who were previously deemed as being unsuitable for surgical resection due to aggressive tumor biology or extensive disease.

Although the concept of “conversion surgery” is not yet uniformly defined in the international HCC field, JLCA recently advocated a definition of “conversion therapy,” using the terms functional conversion and oncological conversion [39, 40]. However, this definition has not yet been globally adopted, and clinical criteria and indications remain heterogeneous across institutions. Nonetheless, growing evidence supports the feasibility of surgical resection after tumor downstaging by systemic therapy in carefully selected patients demonstrating favorable treatment responses.

Among the most pivotal studies, the LENS‐HCC trial, a prospective multicenter phase II study, evaluated the efficacy of lenvatinib as induction therapy followed by surgical resection. Patients received lenvatinib for 8 weeks, after which the resectability was re‐evaluated. A total of 49 patients, mainly those with vascular invasion (65%) and/or extrahepatic metastasis (15%), were enrolled, and the resection rate, the primary endpoint, was 67.3% (33/49) with well‐maintained perioperative safety; the 1‐year survival rate (75.9%) was also favorable. Notably, a resection rate of 76.2% was achieved for tumors initially considered as “oncologically unresectable” in the context of the LENS‐HCC trial—a definition referring to technically resectable tumors with biological high‐risk features such as macroscopic vascular invasion, extrahepatic metastasis, or multinodular disease. In contrast, the resection rate was only 14.3% for those classified as “technically unresectable” [41]. These findings underscore the clinical relevance of biologic resectability, particularly in the context of systemic therapy‐induced tumor shrinkage.

In parallel, the RACB trial, a multicenter prospective study conducted in Japan, explored the combination of atezolizumab plus bevacizumab followed by surgery in patients with initially unresectable HCC. The short‐term outcomes were presented at the 2025 ASCO Gastrointestinal Cancers Symposium. The resection rate was 48%, with R0 resection achieved in 87.5% of the operated cases. Based on the mRECIST criteria, the complete and partial response rates were 2.2% and 26.1%, respectively. Although no treatment‐related deaths were reported, there were cases of perioperative complications such as bile leakage and infections [42, 43].

Furthermore, the LEOPARD‐NEO trial (jRCTs031230128) is currently underway to investigate the feasibility of combining lenvatinib with hepatic arterial infusion chemotherapy (HAIC) followed by resection in patients with vascular invasion but preserved technical resectability. The study aims to define treatment algorithms for patients with locally advanced HCC who might benefit from multimodal treatment strategies.

In addition to these investigator‐initiated trials, another study reported promising results in selected patients who underwent surgical resection after treatment with ICI therapy. This single‐arm phase 1b study evaluated the feasibility of neoadjuvant cabozantinib plus nivolumab in patients with locally advanced HCC. Of the 15 patients enrolled, 12 (80%) underwent margin‐negative surgical resection, and 42% of these patients showed a major pathologic response [44].

These trials suggest that systemic therapy, especially ICI‐based combinations, can alter the biological behaviors of advanced HCC and enable curative resection in selected patients. However, both LENS‐HCC and RACB were single‐arm phase II studies; therefore, they do not demonstrate superiority over other treatment modalities such as continued systemic therapy or locoregional approaches. Accordingly, the optimal timing for surgery, the appropriate duration of preoperative therapy, and the eligibility criteria for response‐based selection remain unresolved. Based on emerging clinical experience, surgery may be most beneficial in patients who achieve sustained tumor control and biological downstaging without radiologic features of aggressive tumor behavior, although this hypothesis requires validation in prospective comparative studies.

**FIGURE 1:**
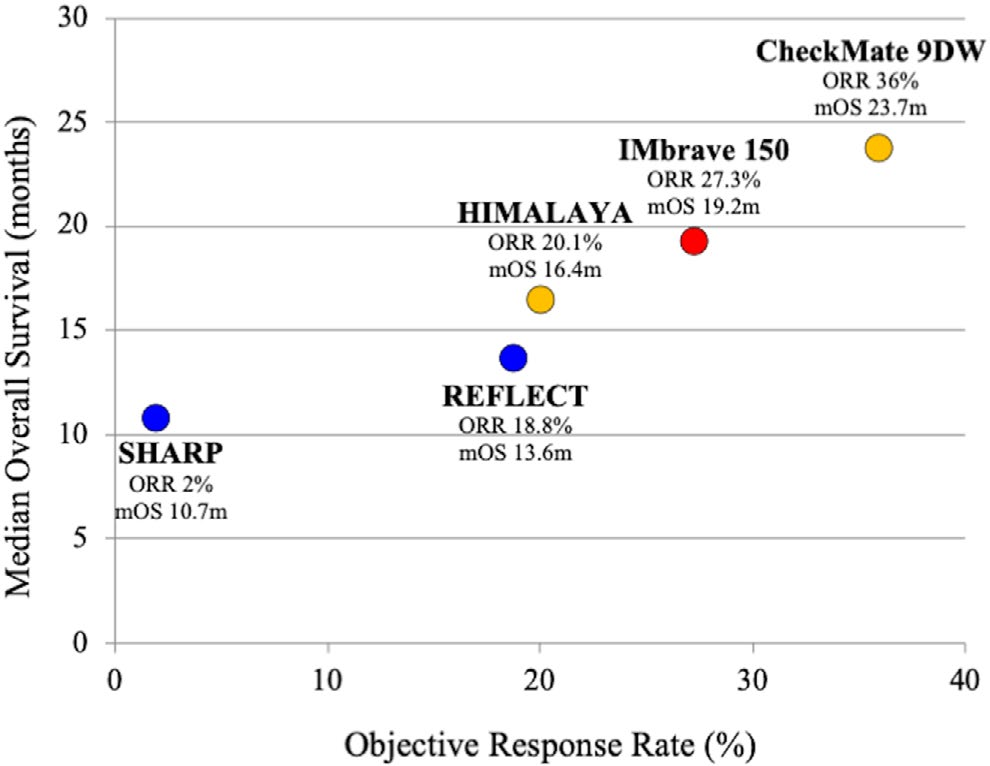
Relationship between the objective response rate (ORR) and median overall survival (OS) across pivotal phase III trials for unresectable hepatocellular carcinoma. Each dot represents a phase III clinical trial evaluating first‐line systemic therapies for unresectable HCC. Blue dots indicate multi‐tyrosine kinase inhibitor (TKI) monotherapies, orange dots represent immune checkpoint inhibitor (ICI) monotherapies plus ICI combination regimens, and red dots represent ICI plus molecular‐targeted agent combination regimens. The ORR values are based on RECIST v1.1. The median OS and ORR are plotted as reported in each clinical trial's publication.

Figure 1 shows a plot of ORR versus median OS across pivotal phase III trials. Higher ORRs and OS were achieved with ICI‐based regimens (e.g., atezolizumab plus bevacizumab, durvalumab plus tremelimumab, nivolumab plus ipilimumab) as compared with TKIs. These results reinforce the notion that achieving radiological and pathological response may facilitate the possibility of additional curative local treatment (Figure 1).

Despite the encouraging signals, guidelines remain cautious. Current guidelines, including the 2021 edition of the Japanese Society of Hepatology (JSH) guideline, do not recommend preoperative therapy for technically resectable HCC as a means to improve the prognosis (Grade C, weak recommendation) [45]. Similarly, the 2024 EASL guidelines advise that neoadjuvant approaches be pursued only within clinical trials until more definitive evidence becomes available [46].

At the same time, caution is warranted regarding perioperative safety after ICI‐based systemic therapy. Although high response rates have renewed enthusiasm for conversion strategies, the incidence of irAEs including treatment‐related deaths reported in recent phase III trials raises important concerns [20]. Evidence remains limited regarding the optimal washout period, preoperative risk stratification, and postoperative management of irAEs, highlighting the need for careful multidisciplinary evaluation when considering resection after ICIs.

Nevertheless, as the landscape continues to evolve with the accumulation of clinical data, future guideline updates may incorporate these emerging strategies as viable treatment options.

## 4 Oncological Resectability Criteria for HCC

Recent advances in systemic therapies for HCC have provoked increased discussions around the appropriate surgical indications within multidisciplinary treatment strategies. However, a lack of consensus on the oncological resectability criteria has precluded the formulation of a clear definition of so‐called “conversion therapy” and hindered constructive discussions on the optimal management of patients with advanced HCC. To provide an objective and reproducible framework for surgical decision‐making in this context, the Japan Liver Cancer Association (JLCA) and the Japanese Society of Hepato‐Biliary‐Pancreatic Surgery (JSHBPS) jointly proposed the oncological resectability criteria for HCC in 2023 [8].

This classification is the result of a rigorous expert panel process that included a comprehensive review of existing literature and clinical data, as well as a nationwide survey of 351 board‐certified expert Japanese hepatobiliary‐pancreatic surgeons [47].

Oncological resectability is classified into the following three categories:

- Resectable (R): Oncological status for which surgery alone may be expected to offer clearly better survival outcomes as compared with other treatments
- Borderline resectable 1 (BR1): Oncological status for which surgical intervention as a part of multidisciplinary treatment may be expected to offer a survival benefit.
- Borderline resectable 2 (BR2): Oncological status initially unsuitable for resection, for which the efficacy of surgery is uncertain, and the surgical indication should be carefully determined under standard multidisciplinary treatments

**FIGURE 2:**
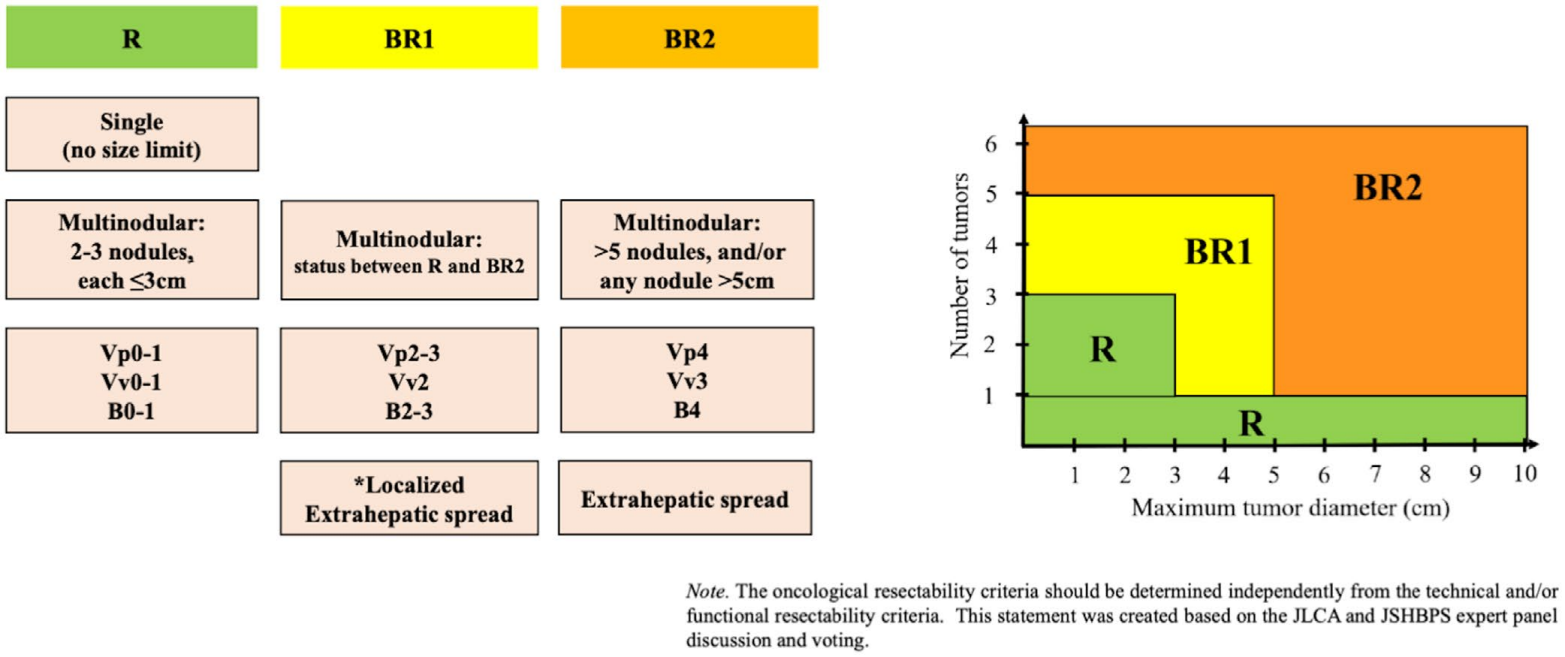
Summary of the proposed oncological resectability criteria. *Localized factors (e.g., solitary nodal involvement of No. 3, 8, or 12 lymph nodes/localized peritoneal dissemination/unilateral adrenal metastasis or oligometastasis to the lung). BR1, borderline resectable 1; BR2, borderline resectable 2; R, resectable. *Source:* Adapted from Akahoshi et al. [8]. This figure is reproduced from an open‐access article distributed under the terms of the Creative Commons Attribution‐NonCommercial (CC BY‐NC 4.0) license (https://creativecommons.org/licenses/by‐nc/4.0/).

Tumor conditions for R, BR1, and BR2 are illustrated in Figure 2. These criteria were developed to maximize the oncological benefits of surgery, under the premise that the surgical resection is feasible based on the technical and liver functional aspects. Thus, these oncological criteria are expected to serve as a common language for discussions and analyses regarding multidisciplinary treatments for HCC and thereby contribute to the construction of future evidence [8].

Although major Western treatment frameworks, such as the BCLC staging system and the EASL Clinical Practice Guidelines, do not yet include a category equivalent to “borderline resectable,” their conceptual foundations show partial complementarity [46]. Whereas the BCLC system typically prioritizes the anticipated oncological benefit of surgery, many Asian guidelines—including APASL and JSH—traditionally emphasize the technical feasibility of resection [45]. The JLCA/JSHBPS oncological resectability criteria bridge these viewpoints by stratifying the expected oncological benefit of surgery in the era of highly effective systemic therapies, thereby offering a framework that may complement global treatment algorithms.

### 4.1 Validation Studies of the Oncological Resectability Criteria

Since the proposal of the oncological resectability criteria for HCC, several retrospective studies have attempted to validate their clinical usefulness. Shindoh et al. first demonstrated the prognostic utility of the oncological resectability criteria in a retrospective analysis of 1822 patients who underwent curative‐intent resection at a single institution. Multivariate analysis confirmed that classification into BR1 and BR2 categories was significantly associated with both the OS (BR1: HR, 1.88; 95% CI, 1.38–2.55; BR2: HR, 4.12; 95% CI, 3.01–5.65) and recurrence‐free survival (BR1: HR, 1.86; 95% CI, 1.44–2.41; BR2: HR, 3.62; 95% CI, 2.71–4.82) versus the resectable category [48]. These findings lent support to the usefulness of the criteria for stratifying the long‐term outcomes following surgical resection.

Komatsu et al. subsequently evaluated the clinical validity of the oncological resectability criteria using a large retrospective dataset consisting of 931 patients who underwent curative‐intent hepatectomy and 273 patients who received systemic therapy for unresectable HCC. Patients were classified into R, BR1, and BR2 categories, and the OS could also be significantly stratified across the three groups (median OS: R, 107.2 months; BR1, 44.4 months; BR2, 18.4 months; *p* < 0.0001). Notably, among patients classified as BR2, the survival of those who underwent resection was comparable to that of those who received systemic therapy, suggesting no clear survival benefit from surgery in this group [49]. These findings further reinforce the oncological relevance and real‐world applicability of the oncological resectability criteria.

Shindoh et al. further examined the prognostic impact of preoperative systemic therapy in a multi‐institutional cohort of patients who underwent liver resection for HCC. Notably, among patients with BR1 tumors, those who received preoperative systemic therapy exhibited significantly better disease‐specific and recurrence‐free survival as compared with those who underwent upfront surgery. In contrast, no clear survival benefit was observed in the BR2 group [50]. These findings suggest that the oncological resectability criteria can help guide patient selection for preoperative systemic therapy within the framework of a multidisciplinary treatment strategy.

Furthermore, an international multi‐institutional study externally validated the oncological resectability criteria in a global cohort of 1766 patients who underwent curative‐intent hepatectomy for HCC. The original oncological resectability criteria (R, BR1, BR2) allowed significant stratification of the OS. Importantly, the investigators proposed a refined TBS‐BR model by substituting categorical tumor size and number with the continuous tumor burden score (TBS). This revised model, when combined with patient factors such as the ASA class, ALBI score, and serum AFP, provided superior prognostic discrimination as compared to both the original oncological resectability criteria and the BCLC system. These findings not only confirm the international applicability of the BR‐HCC framework but also underscore the potential usefulness of TBS‐based refinements to further delineate the BR subgroup and guide future updates of the criteria [51].

Beyond serving as a framework for assessing surgical indications and predicting postoperative prognosis, the oncological resectability criteria have the potential to guide the integration of systemic therapy with surgery within multidisciplinary treatment strategies. However, current validation has been primarily retrospective, and their utility in treatment decision‐making has not yet been conclusively demonstrated. Therefore, future prospective trials and real‐world registries designed around the resectability framework are essential to determine whether the criteria can inform the optimal timing, sequencing, and patient selection for surgery following systemic therapy.

## 5 Conclusions

The treatment landscape for HCC has undergone a paradigm shift driven by novel systemic therapies, particularly ICI‐based combinations, enabling multidisciplinary strategies beyond traditional locoregional approaches. Within this context, the oncological resectability criteria proposed by the JLCA and JSHBPS provide an objective framework for evaluating surgical indications in the era of effective systemic therapy, and their prognostic validity has been consistently demonstrated in external cohorts.

Looking ahead, more evidence is needed to establish the clinical utility of these criteria for treatment decision‐making. Prospective, criteria‐based trials and real‐world registries will be essential for defining standardized timing and sequencing of resection, refining eligibility assessment (including the role of radiologic and biological responses), and identifying which patient subgroups gain the most oncological benefit. Ultimately, these efforts might support integrating resectability‐based strategies into international treatment guidelines and contribute to personalized, evidence‐based care for patients with advanced HCC.

## Author Contributions

**Keiichi Akahoshi:** funding acquisition, writing – original draft, writing – review and editing, visualization, methodology, data curation, conceptualization, investigation. **Shun Kaneko:** writing – review and editing, conceptualization, validation, investigation. **Shinji Tanaka:** funding acquisition, writing – review and editing, validation. **Minoru Tanabe:** conceptualization, investigation, writing – review and editing, writing – original draft, supervision, project administration. **Daisuke Ban:** supervision, writing – review and editing, validation, methodology, conceptualization.

## Funding

This work was supported by Program for Basic and Clinical Research on Hepatitis (JP25fk0210136) from Japan Agency for Medical Research and Development (AMED), JSPS KAKENHI Grant Numbers 19K2390 and 25K06488.

## Conflicts of Interest

Keiichi Akahoshi, Shun Kaneko, and Minoru Tanabe declare no conflicts of interest. Shinji Tanaka and Daisuke Ban are Editorial Board Members of Annals of Gastroenterological Surgery. The authors declare that this does not influence the content or conclusions of this manuscript.

## Supporting information


**Table S1:** ags370153‐sup‐0001‐TableS1.pdf.
