# Supplementary material for: Current Status of Multidisciplinary Treatment Strategies for Hepatocellular Carcinoma in the Era of Advanced Systemic Therapies
Source: Ann Gastroenterol Surg. 2025 Dec 22;10(2):325–35. doi: 10.1002/ags3.70153 (PMC12962028; doi:10.1002/ags3.70153)
Supplement: Supplementary file 1 — Table S1: ags370153‐sup‐0001‐TableS1.pdf. [file AGS3-10-325-s001.pdf]

Supplemental Table 1 Response outcomes of pivotal phase III clinical studies of systemic therapies for hepatocellular carcinoma

Trial	SHARP		REFLECT		IMbrave 150		HIMALAYA		CheckMate 9DW	
Response rate (%) (RECIST 1.1/ mRECIST)	Sorafenib	Placebo	Lenvatinib	Sorafenib	Atezolizumab-Bevacizumab	Sorafenib	STRIDE	Sorafenib	Nivolumab plus ipilimumab	Lenvatinib or Sorafenib
Complete response	0/ NR	0/ NR	0.4/ 2	0.2/ 1	5.5/ 10.2	0/ 1.9	3.1/ NR	0/ NR	7/ NR	2/ NR
Partial response	2/ NR	1/ NR	18/ 38	6/ 12	21.8/ 23.1	11.9/ 11.4	17/ NR	5.1/ NR	29/ NR	11/ NR
Stable disease	71/ NR	67/ NR	54/ 33	53/ 46	46.3/ 39.1	43.4/ 41.8	39.9/ NR	55.5/ NR	32/ NR	62/ NR
Progressive disease	NR	NR	18/ 17	32/ 32	19.6/ 20.3	24.5/ 25.3	39.9/ NR	39.3/ NR	20/ NR	14/ NR
Objective response rate	2/ NR	1/ NR	18.8/ 40.6	6.5/ 12.4	27.3/ 33.2	11.9/ 13.3	20.1/ NR	5.1/ NR	36/ NR	13/ NR
Disease control rate	43/ NR	32/ NR	72.8/ 73.8	59/ 58.4	73.6/ 72.3	55.3/ 55.1	60.1/ NR	60.7/ NR	68/ NR	75/ NR

In the SHARP trial, PD was not explicitly reported, and the definition of disease control rate (DCR) required that CR, PR, or SD be maintained for ≥28 days, which differs from current trial reporting standards.
NR: Not reported
